# Cigarette Smoke Induces Intestinal Inflammation via a Th17 Cell-Neutrophil Axis

**DOI:** 10.3389/fimmu.2019.00075

**Published:** 2019-01-29

**Authors:** Myunghoo Kim, Bonhee Gu, Matthew C. Madison, Hyo Won Song, Kendra Norwood, Andrea A. Hill, Wan-Jung Wu, David Corry, Farrah Kheradmand, Gretchen E. Diehl

**Affiliations:** ^1^Department of Molecular Virology and Microbiology, Alkek Center for Metagenomics and Microbiome Research, Baylor College of Medicine, Houston, TX, United States; ^2^Departments of Pathology and Immunology, Baylor College of Medicine, Houston, TX, United States; ^3^Biology of Inflammation Center, Baylor College of Medicine, Houston, TX, United States; ^4^Department of Medicine, Pulmonary and Critical Care, Baylor College of Medicine, Houston, TX, United States; ^5^The Dan L. Duncan Cancer Center, Baylor College of Medicine, Houston, TX, United States; ^6^Center for Translational Research in Inflammatory Diseases, Michael E. DeBakey VA Medical Center, Houston, TX, United States

**Keywords:** cigarette smoke, intestinal inflammation, Th17 cells, neutrophils, lung-gut axis

## Abstract

Epidemiological evidence finds cigarette smoking is a common risk factor for a number of diseases, not only in the lung but also in other tissues, such as the gastrointestinal tract. While it is well-documented that smoking directly drives lung inflammatory disease, how it promotes disease in peripheral tissues is incompletely understood. In this study, we utilized a mouse model of short-term smoke exposure and found increased Th17 cells and neutrophilia in the lung as well as in the circulation. Following intestinal inflammatory challenge, smoke exposed mice showed increased pathology which corresponds to enhanced intestinal Th17 cells, ILC3 and neutrophils within intestinal tissue. Using cellular depletion and genetic deficiencies, we define a cellular loop by which IL-17A and downstream neutrophils drive cigarette smoke-enhanced intestinal inflammation. Collectively, cigarette smoke induced local lung Th17 responses lead to increased systemic susceptibility to inflammatory insult through enhanced circulating neutrophils. These data demonstrate a cellular pathway by which inflammatory challenge in the lung can sensitize the intestine to enhanced pathological innate and adaptive immune responses.

## Introduction

Cigarette smoke (CS) is the major preventable cause of human death (1). Despite public awareness of the harmful effects of smoking, there is increased prevalence of smoking in many developing countries (2). CS or components of CS directly impact lung immune homeostasis leading to fatal illness including chronic obstructive pulmonary disease and lung cancer (3, 4). CS exposure also impacts immune-mediated diseases in other tissues, such as cerebrovascular (3) and cardiovascular diseases (4). Within the intestine, CS influences the severity of inflammatory bowel disease (IBD) in a way that is incompletely understood. For example, epidemiological evidence demonstrates that smoking is the most important environmental risk factor for Crohn's disease (5). In contrast, nicotine, a major component of CS, is thought to play an anti-inflammatory role in ulcerative colitis (6). Together, the underlying immune cellular mechanisms by which CS affects pathogenesis of intestinal inflammatory diseases are unknown.

The lung and intestinal tracts are both mucosal tissues and, in the context of immunity to pathogens, there is evidence that lung and intestinal immunity can influence each other (7). However, many critical questions remain as to how local tissue immunity alters immune response in distal tissue sites. Given the association between smoking and intestinal inflammation, we wanted to determine the impact of altered lung immune responses on the regulation of intestinal inflammation.

Intestinal inflammation can be induced by a variety of environmental factors including pathogen infection, toxins, and allergen exposure. Intestinal inflammation is mediated by the accumulation of innate immune cells and expansion of pro-inflammatory T cells (8). In response to inflammatory signals, significant numbers of circulating neutrophils and monocytes accumulate in the inflamed tissue and can amplify inflammatory responses. T cells respond to local cues including cytokine production by antigen presenting cells to differentiate into effector T cells, such as Th1 and Th17 cells which can further mediate intestinal inflammatory diseases (8, 9). Chronic intestinal inflammation, such as IBD is a major risk factor for development of cancer with in the gastrointestinal tract including colorectal cancer (10, 11). Therefore, understanding how environmental contribute to an inflammatory environment will enable development of therapeutics to limit tissue damage and cancer development.

Using a mouse model of CS exposure (12, 13), we established that short term exposure results in an altered lung inflammatory milieu without tissue pathology, such as emphysema, allowing us to determine the impact of CS exposure on intestinal inflammation in the absence of secondary effects due to lung tissue damage. In short term CS exposed mice, we found an increased Th17 signature in the lung. We further identified increased pro-Th17 cytokines and neutrophil numbers in the blood circulation after CS exposure. To determine the role of CS in inducing intestinal inflammation, we treated CS exposed mice with dextran sodium sulfate (DSS) to induce intestinal damage and found CS-exposed mice had enhanced intestinal inflammation with elevated Th17 cell, ILC3, and neutrophil responses in the intestine. By utilizing strategies for selective depletion or cytokine deficiency, we further demonstrated that this IL-17A-neutrophil axis is key to enhancing intestinal inflammation after CS exposure. These data demonstrate crosstalk between organ specific immune responses where environmental priming of a lung inflammatory response leads to enhanced systemic innate responses that increase susceptibility to intestinal inflammatory insult.

## Materials and Methods

### Mice

C57BL/6J (Jax # 000664) and IL-17a^−/−^ mice [C57BL/6 background, obtained from Dr. Chen Dong (The University of Texas MD Anderson Cancer Center, Houston, TX)] were kept in specific pathogen-free conditions and bred at the animal facility of Baylor College of Medicine. Mouse experiments were performed with at least three female mice per group (6–8 weeks of age). Littermate mice were randomly assigned to experimental groups. All animal experiments were performed in accordance with approved protocols for the BCM Institutional Animal Care and Usage Committee.

### Cigarette Smoke Exposure

Mice were exposed to cigarette smoke as previously described (12, 13). Briefly, 6-weeks-old female mice were exposed to active smoke from commercial cigarettes (Marlboro 100's). Exposure to 3 cigarettes (~4–5 min per cigarette) per day, 5 days a week for 2 months was carried out by intermittently forcing air (4 l min^−1^) through the burning cigarette. Intermittent cycles were designed to mimic puffing cycles of human smokers and to prevent CO_2_-induced asphyxiation. Puffing cycles consisted of 5 s of active cigarette smoke followed by 25 s of forced air by a timer controlled two-way valve (Humphrey, Kalamazoo, MI). Mice were given 10 min of rest between each cycle of cigarette smoke exposure. For nicotine exposure, mice were intranasally (i.n.) administered 2.5 ug of nicotine once per day, 5 days a week.

### DSS-Induced Intestinal Inflammation

For intestinal inflammation, mice were provided 2% DSS in drinking water for 6 days followed by plain water before analysis on day 12. Colon tissues were processed to assess for histological changes. Colonic or lung tissues were examined by flow cytometry for T cells, ILCs, neutrophils and by qRT-PCR for expression of inflammatory cytokines and neutrophil responses. Blood and lung homogenate were collected. Cytokine concentrations were examined by Luminex assay according to manufacturer's protocol (Millipore). When indicated, mice were injected intraperitoneally with antibodies (50 mg/injection for each antibody) to deplete neutrophils (anti-Ly6G or isotype (BioXcell) on day −2, 0, 2 post-DSS challenge). As control animals, mice were injected with isotype antibody.

### Cell Isolation

Large intestinal lamina propria cells were isolated as previously described (14). Briefly, mouse intestines were washed in PBS, once with 1 mM DTT and twice with 30 mM EDTA, and then digested in 100 U/ml type VIII collagenase (Sigma-Aldrich) and 150 ug/ml DNase-containing media with 10% fetal bovine serum. Digested material was passed through a cell strainer and separated on a discontinuous 40/80% Percoll gradient. Mouse immune cell isolation from lung and mesenteric lymph node (MLN) were prepared by mechanical disruption, filtered through a 40-um Falcon cell strainer, followed by RBC lysis (ACK lysis buffer, Sigma) for 3 min.

### Antibodies, Cell Staining, and Flow Cytometry

Flow cytometric analysis was performed on an LSR II (BD Biosciences) and analyzed using FlowJo software (Tree Star Inc.). Antibodies were from BD Pharmingen, eBiosciences, or BioLegend. DAPI or UV live/dead fixable dead cell stain (ThermoFisher) was used to exclude dead cells. Total cell counts were determined with 123count eBeads (ThermoFisher). For intracellular staining of cytokines, cells were activated in RPMI 1640 (10% fetal bovine serum) with phorbol myristate acetate (50 ng/ml), ionomycin (1 μM), and GolgiPlug (BD bioscience) for 4 h. Cells were then stained for surface antigens [CD3 (145-2C11), TCRβ (H57-597), and/or CD4 (RM4-5)]. Cells were then fixed, permeabilized, and stained with antibodies to IL-17A (TC11-18H10.1), IL-22 (IL-22JOP), or IFN-γ (XMG1.2). Cells were stained with an antibody to mouse FoxP3 (FJK-16s), T-bet (4B10), GATA-3 (16E10A23), or RORγt (BD2) according to the manufacturer's protocol (eBioscience). For neutrophils, cells were stained with antibodies for CD11b (M1/70), Ly6C (AL-21), and Ly6G (1A8). For ILCs, cells were stained with Lineage antibody cocktail, CD90.2 (53-2.1) then permeabilized and stained for intracellular T-bet, GATA-3, or RORγt.

### RNA Extraction and Real-Time RT-PCR

RNA from MLN, lung and colon tissue was prepared with Trizol (Invitrogen) according to manufacturer's instructions. RNA was reverse transcribed into cDNA (SuperScript III; Invitrogen) and qPCR was performed with Applied Biosystems Viia7 Real-time PCR system with SYBR Green Supermix (Roche), 20 pmol forward and reverse primers, and 0.1 μg of cDNA. The thermocycling program was 40 cycles at 95**°**C for 15 s, 60**°**C for 30 s, and 72**°**C for 30 s, with an initial cycle of 95**°**C for 2 min. Relative levels of target gene were determined by using the delta Ct value compared to delta Ct (GAPDH). qRT-PCR was performed with primers described previously (15) or as follows:

**Table:** 

**mIL-10-F**	**CATCATGTATGCTTCTATGCAG**
**mIL-10-R**	**CCAGCTGGACAACATACTGCT**
**GAPDH-F**	**ACCACAGTCCATGCCATCAC**
**GAPDH-R**	**TCCACCACCCTGTTGCTGT**
**IL-23-F**	**CCAGCAGCTCTCTCGGAATC**
**IL-23-R**	**GATTCATATGTCCCGCTGGTG**
**MPO-F**	**TCCCACTCAGCAAGGTCTT**
**MPO-R**	**TAAGAGCAGGCAAATCCAG**
**Elastase-F**	**GTTGGGCACAAACAGACC**
**Elastase-R**	**GCAAACTCAGCCACAGG**
**MMP9-F**	**ATAGAGGAAGCCCATTACAGG**
**MMP9-R**	**GTGTACACCCACATTTGACG**

### Histopathology

Tissues were fixed in 10% neutral buffered formalin, routinely processed, sectioned at 6 um, and stained with hematoxylin and eosin (H&E) for light microscopic examination. Samples were assessed in a blinded fashion. Samples were scored using a standard scoring system of 0–4 based on previously described criteria (16).

### Statistical Analysis

To determine if data followed a normal distribution we performed Shapiro-Wilk test. If normality was not rejected, we utilized Student's *t*-test for two groups or two-way ANOVA with Bonferroni's correction. If normality was rejected, we utilized Mann-Whitney test for two independent groups and Kruskal-Wallis test with Dunn's multiple comparison post-test for more than two groups. All analyses were performed using GraphPad Prism version 7.0. Differences were considered to be significant at ns: *P* > 0.05, ^*^P ≤ 0.05, ^**^P < 0.01, ^***^P < 0.001.

## Results

### Cigarette Smoke Exposure Increases Intestinal Inflammation

**Figure 1:**
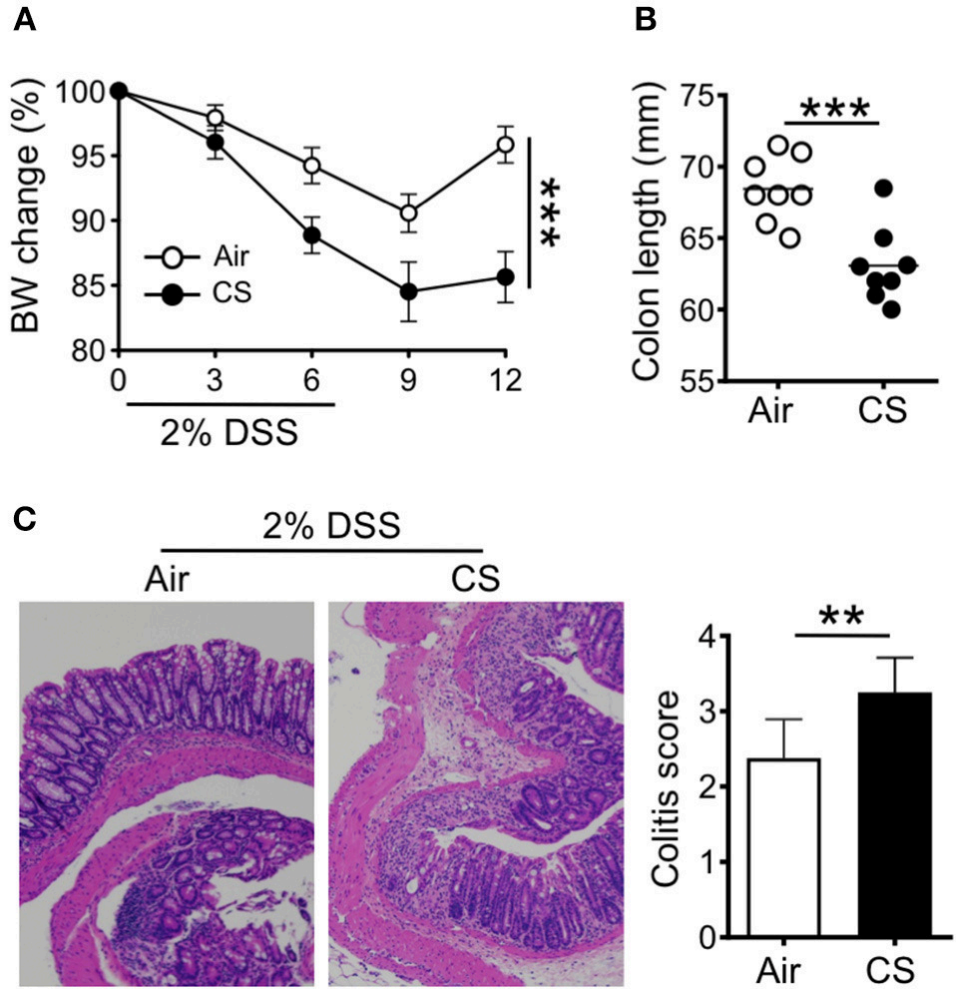
Cigarette smoke promotes intestinal inflammation. **(A–C)** Mice were exposed to air or cigarette smoke (CS) for 2 months before treatment with 2% DSS for 6 days. Body weight changes **(A)**, colon length (mm) **(B)**, and representative H&E staining of colon tissue and colitis scores **(C)**. Representative and pooled data obtained (mean ± SEM, *n* = 8–9) from two independent experiments are shown. Student's *t*-test **(A,B)**, Mann-Whitney **(C)** was used to determine significance. ^**^P < 0.01; ^***^P < 0.001.

We first sought to determine the impact of CS on intestinal inflammation. To this end, we employed our well-established mouse model of smoke exposure (12, 13) in combination with the DSS induced-colitis model. Mice were treated with air or CS for 2 months before treatment with 2% DSS for 6 days. Compared to air exposed mice, CS exposed mice exhibited increased weight loss with delayed recovery after DSS treatment (Figure 1A) as well as shorter colon length (Figure 1B). We found increased histological damage in CS mice including disrupted villi structure, and increased immune cell infiltration including neutrophils, together resulting in higher colitis scores (Figure 1C). Nicotine is a major component of cigarette that is known to have immune-modulatory effects (6). We treated mice with nicotine alone, but found no impact on intestinal inflammation in response to DSS treatment (Supplementary Figures 1A,B). Overall, these results indicate that CS exposure increases intestinal damage after inflammatory challenge.

### CS Induces Lung Th17 Differentiation With Increased Pro-Th17 Cytokines and Neutrophils in the Lung

**Figure 2:**
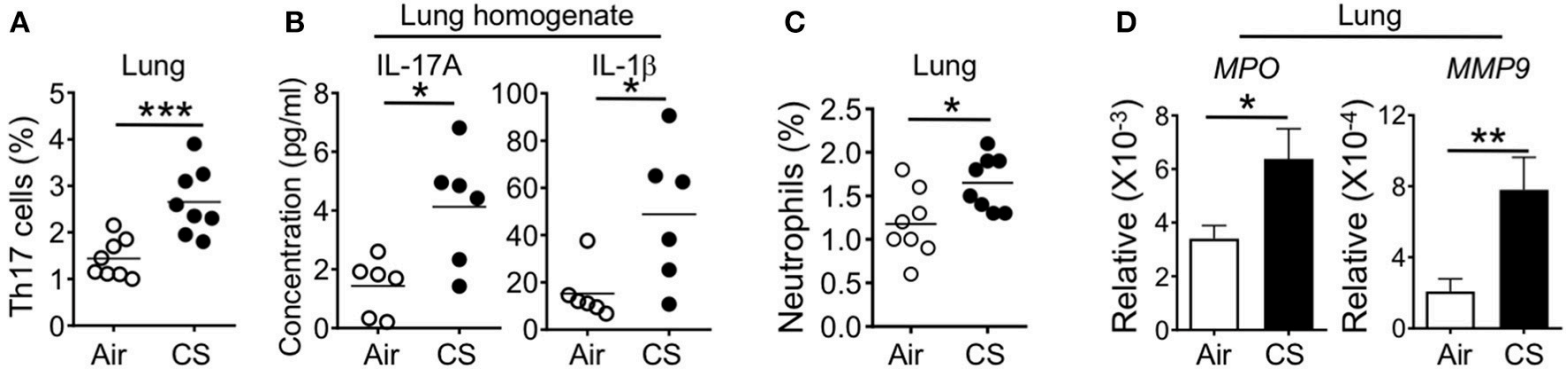
Cigarette smoke induces lung Th17 cell and neutrophil responses. Mice were exposed to air or CS for 2 months. **(A)** Percentages of RORγt^+^ cells among lung CD3^+^TCRβ^+^CD4^+^ T cells. **(B)** Cytokines in lung homogenates as determined by Luminex assay. **(C)** Frequencies of neutrophils (CD11b^+^Ly6G^+^ cells) in the lung of mice exposed to air or CS for 2 months. **(D)** Lung mRNA expression of neutrophil response genes as determined by qRT-PCR. Data is representative of two independent experiments with 6–8 animals per group (mean ± SEM). Student's *t*-test **(A,C,D)** or Mann-Whitney test **(B)** was used to determine significance. ^*^P ≤ 0.05; ^**^P < 0.01; ^***^P < 0.001.

To understand how CS exposure resulted in heightened sensitivity to DSS colitis, we analyzed whether CS exposure altered immune responses in the lung. We and other groups previously reported that long term CS exposure induced multiple immune alterations to the lung immune system (12, 13). This includes activation of lung antigen presenting cells (APC) in human patients and mice. In mouse models, this activation promotes Th17 cell differentiation that drives an emphysema-like lung pathology after long-term exposure (12). In our experiments, 2 months of smoke exposure is not sufficient to induce an emphysema phenotype with 4 months of smoke exposure required (12). However, after 2 months of CS exposure we observed increased Th17 cell frequency in the lung and secretion of IL-17A in lung homogenates (Figures 2A,B). We also found increased production in the lung of the pro-Th17 cytokine IL-1β (Figure 2B). Accumulating evidence suggests crosstalk between neutrophils and Th17 cells induces local tissue inflammation which can lead to tissue damage after microbial infection or tissue injury (17). As expected, CS-exposed mice had increased frequencies of neutrophils in the lung (Figure 2C). Neutrophils produce peroxidase and proteolytic enzymes, which mediate inflammatory diseases (18). In accordance with an elevated neutrophil pool in the lung tissue, we also found increased lung expression of the neutrophil activity-associated factors myeloperoxidase (MPO) and matrix metalloproteinase-9 (MMP9) after CS exposure (Figure 2D).

### CS-Exposure Alone Is Not Sufficient to Enhance Intestinal Th17 Cell Responses

**Figure 3:**
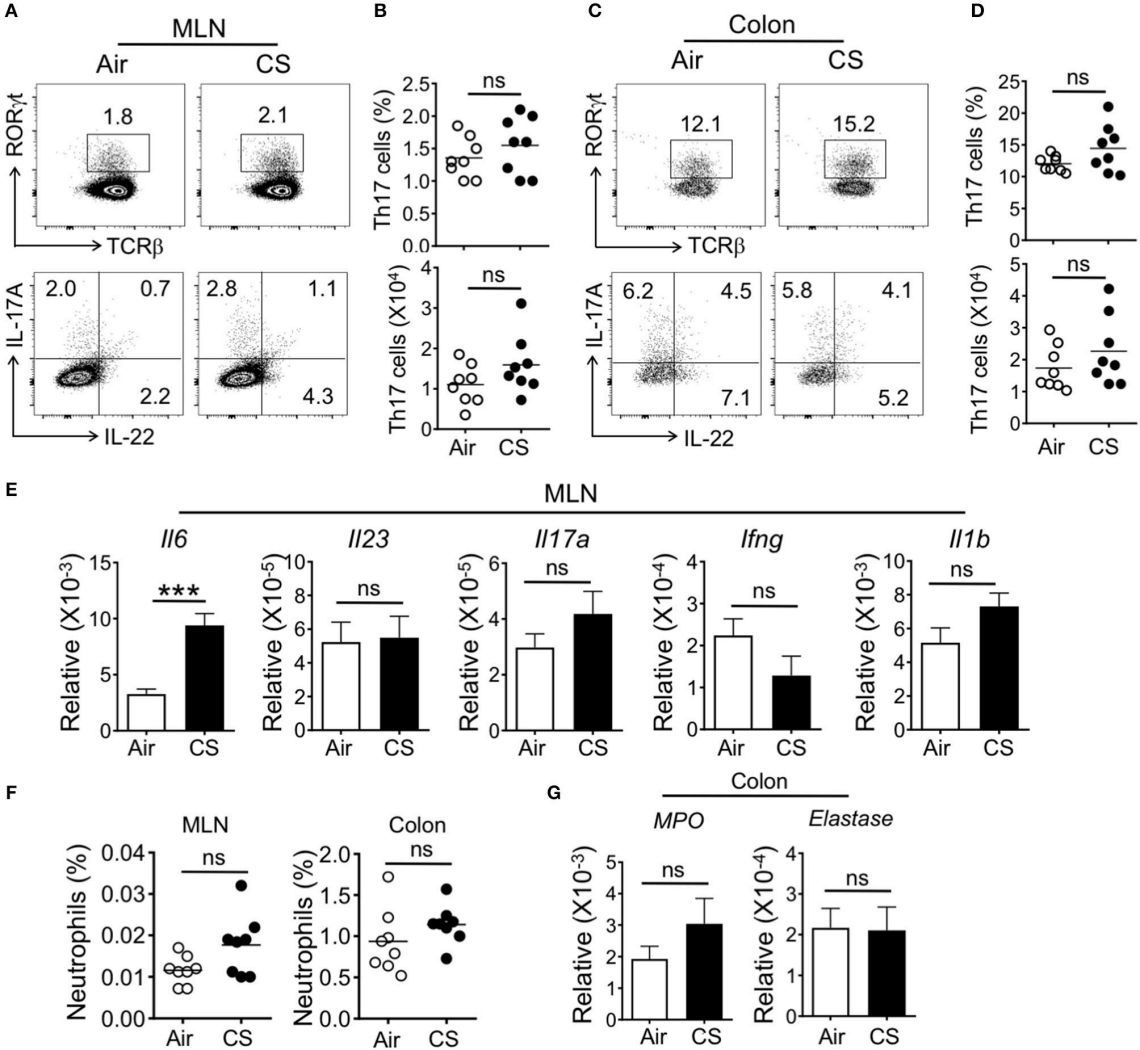
CS exposure alone does not enhance intestinal Th17 cell and neutrophil responses. Mice were exposed to air or CS for 2 months. **(A–D)** Frequencies (%) and absolute numbers of Th17 cells (gated on CD3^+^CD4^+^) in mesenteric lymph node (MLN) and colonic lamina propria as determined by flow cytometry. **(A,C)** Representative dot plots are shown of total Th17 cells (CD3^+^CD4^+^TCRβ^+^RORγt^+^ cells, top) and IL-17A expressing Th17 cells (CD3^+^CD4^+^IL-17A^+^ cells, bottom). **(B,D)** Frequency and absolute cell numbers of IL-17A producing cells (CD3^+^CD4^+^IL-17A^+^ cells). **(E)** mRNA expression as determined by qRT-PCR for cytokines in MLN from Air and CS mice. **(F)** Frequencies of neutrophils (CD11b^+^Ly6G^+^ cells) in MLN and colon. Percentages of CD11b^+^Ly6G^+^ neutrophils among live cells are shown. **(G)** mRNA expression as assessed by qRT-PCR for neutrophil response genes in the colon tissues from Air and CS mice. Data is representative of two independent experiments with eight animals per group (mean ± SEM). Student's *t*-test was used to determine significance. ns, *P* > 0.05; ^***^P < 0.001.

We next checked if CS-exposed mice had enhanced intestinal Th17 cell or neutrophil responses. In the colonic lamina propria and mesenteric lymph node (MLN), we failed to observe altered T cell responses, including Th17 or Th22 cells or ILC populations (Figures 3A–D and Supplementary Figures 2, 3). However, we found increased mRNA expression of the pro-Th17 cytokine IL-6 in the MLN with no alterations in other inflammatory cytokines (Figure 3E). Along with the lack of intestinal Th17 cell responses, we did not detect increased MLN or intestinal neutrophils or neutrophil activity-associated factors in the intestine (Figures 3F,G).

### CS-Exposed Mice Display Systemic Inflammation and Neutrophilia

**Figure 4:**
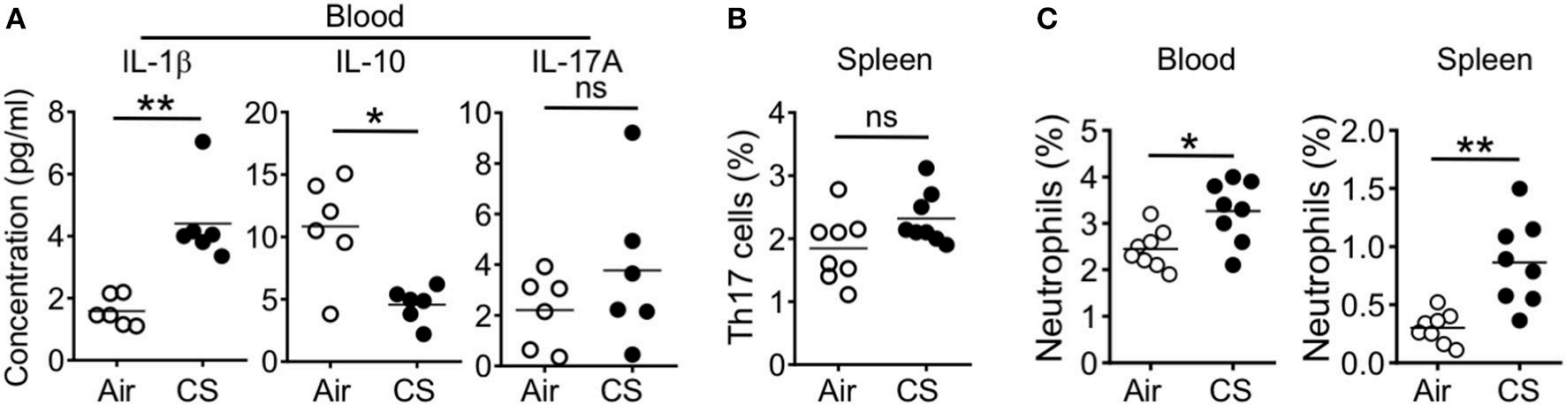
CS exposure induce systemic pro-Th17 cell responses and neutrophilia. Mice were exposed to air or CS for 2 months. **(A)** Quantification of indicated cytokines in the blood as determined by Luminex assay **(B)** Frequencies of Th17 cells in spleen from Air and CS mice as determined by flow cytometry. **(C)** Frequencies of neutrophils (CD11b^+^Ly6G^+^ cells) in blood and spleen from air or CS mice. Percentages of CD11b^+^Ly6G^+^ neutrophils among live cells are shown. Data is representative from two independent experiments with 6–8 animals (mean ± SEM). Mann-Whitney test **(A)** or Student's *t*-test **(B,C)** was used to determine significance. ns, *P* > 0.05; ^*^P ≤ 0.05; ^**^P < 0.01.

When we analyzed the blood and spleen of CS exposed mice, we found systemic effects of smoke exposure. After 2 months of CS exposure and in parallel with observed lung alterations, we found increased blood concentration of the pro-Th17 cytokine IL-1β and decreased concentration of the anti-inflammatory cytokine IL-10 (Figure 4A). However, we did not observe increased levels of IL-17A in the blood and further found no increase in splenic Th17 cell frequencies (Figures 4A,B). We did find increased neutrophil frequency in the blood and spleen (Figure 4C).

Together, our immune profiling shows increased Th17 cell responses in the lung with increased downstream effectors including inflammatory cytokines and neutrophils in systemic sites including blood and spleen. However, CS-exposure alone did not directly lead to accumulation of inflammatory cells within intestinal tissue.

### Increased Th17 Cell, ILC3, and Neutrophil Responses in the Intestine of CS-Exposed Mice During DSS-Colitis

**Figure 5:**
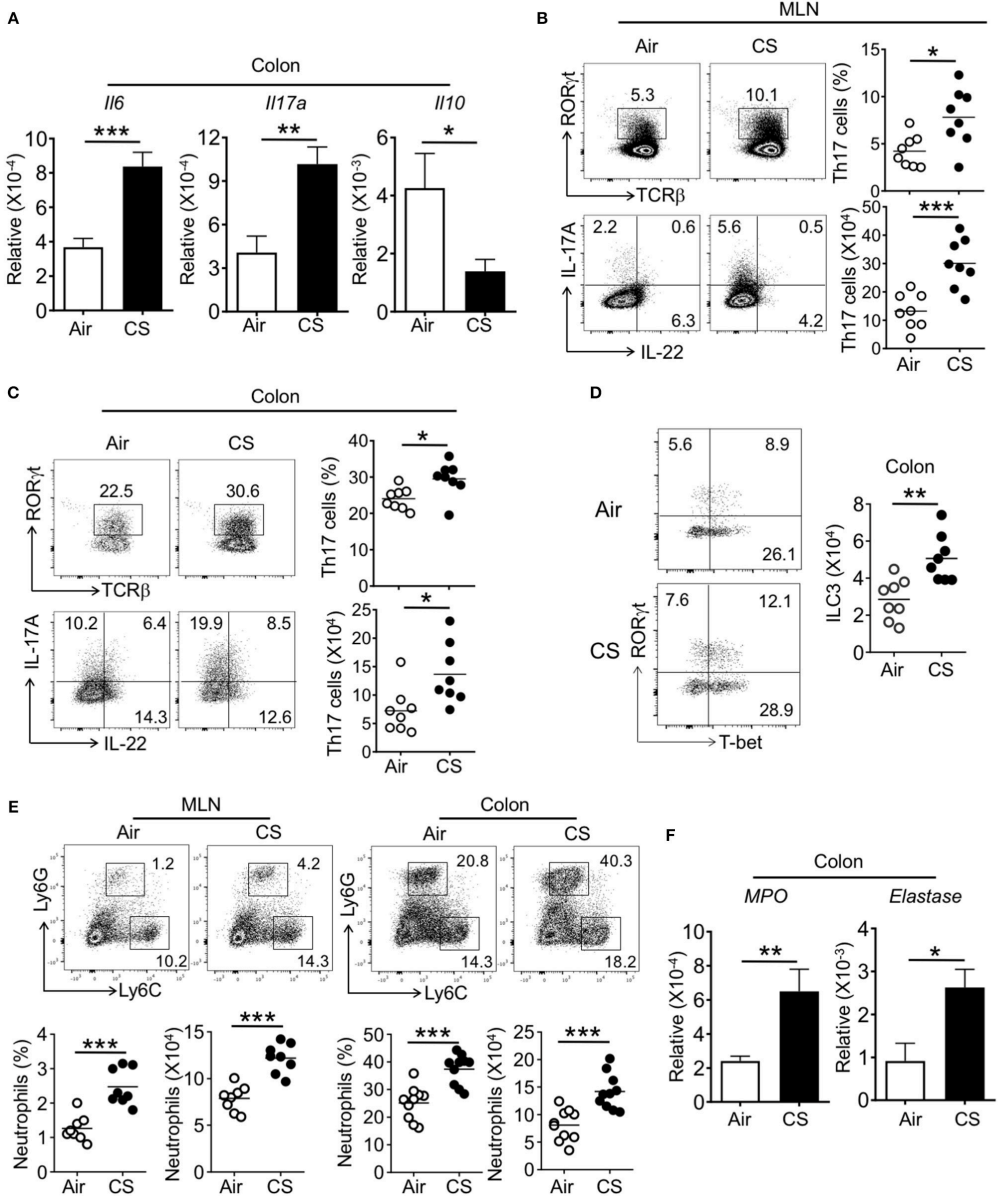
Cigarette smoke exposed mice display elevated intestinal Th17 cell and neutrophil responses after DSS challenge. Mice were exposed to air or CS for 2 months followed by 2% DSS for 6 days. Mice were sacrificed at 9 days post-DSS treatment. **(A)** mRNA expression for indicated cytokine in the colon from DSS treated Air and CS mice as determined by qRT-PCR. **(B,C)** Frequencies (%) and absolute numbers of Th17 cells in MLN and colonic lamina propria from DSS treated Air and CS mice as determined by flow cytometry. Representative dot plots are shown and frequencies and numbers of IL-17A^+^ Th17 cells (CD3^+^CD4^+^IL-17A^+^ cells). **(D)** Representative dot pots and absolute number of ILC3 (Lineage^−^CD90^+^RORγt^+^ cells) in colonic lamina propria from DSS treated Air and CS mice. Cells shown are gated as lineage^−^CD90^+^ cells. **(E)** Frequencies of neutrophils in MLN and colonic lamina propria from Air and CS mice after DSS challenge. Frequencies are % of Ly6G^+^ cells among CD11b^+^ cells. **(F)** mRNA expression for neutrophil response genes in the colon tissues from DSS treated Air and CS mice as determined by qRT-PCR. Data is representative or pooled data from two independent experiments with 8–10 animals (mean ± SEM). Student's *t*-test was used to determine significance. ^*^P ≤ 0.05; ^**^P < 0.01; ^***^P < 0.001.

We next assessed intestinal immune responses in CS exposed mice during DSS-induced inflammation. Within the colon, CS/DSS mice had increased IL-6 and IL-17A gene expression alongside decreased IL-10 expression (Figure 5A). Supporting the increased observed intestinal pathology, CS/DSS mice showed increased Th17 cell frequency and number in the MLN and colon as compared to Air/DSS mice (Figures 5B,C). We found no differences in Th22 cell frequency between Air and CS mice treated with DSS (Figures 5B,C and Supplementary Figure 2). Further, the proportion of other T cell subsets (Th1, Th2, and Treg) in the spleen, MLN, or intestine were comparable between Air and CS treatment after DSS treatment (Supplementary Figure 2). Parallel to increased Th17 cells, we found increased numbers of type 3 innate lymphoid cells (ILC3, Lineage^−^CD90^+^RORγt^+^ cells) in the colon of CS treated mice after DSS challenge (Figure 5D). Like the changes in T cell subsets, other ILC subsets (Lineage^−^CD90^+^T-bet^+^ ILC1, Lineage^−^CD90^+^Gata3^+^ ILC2) were not altered after CS exposure (Supplementary Figure 3). Overall, we observed amplified Th17 and ILC3 responses after DSS challenge in the intestines of CS exposed mice.

After DSS challenge, a number of innate cell populations are known to increase in the intestine that contribute to pathology, including neutrophils (19). In parallel to finding of increased neutrophils in the lung after CS exposure, we also found increased neutrophil recruitment and related activity genes expression, such as *MPO* and *elastase* in the intestines of DSS treated mice exposed to CS (Figures 5E,F). In the blood, CS-exposed, DSS treated mice (CS/DSS) displayed an increased pro-Th17 and IL-17A response (Supplementary Figure 4). These data indicate that CS sensitizes the host immune system to amplify Th17 cells, ILC3, and neutrophil responses in the intestine during colitis.

### CS-Driven IL-17A Responses Mediates Neutrophil Recruitment and Intestinal Inflammation

**Figure 6:**
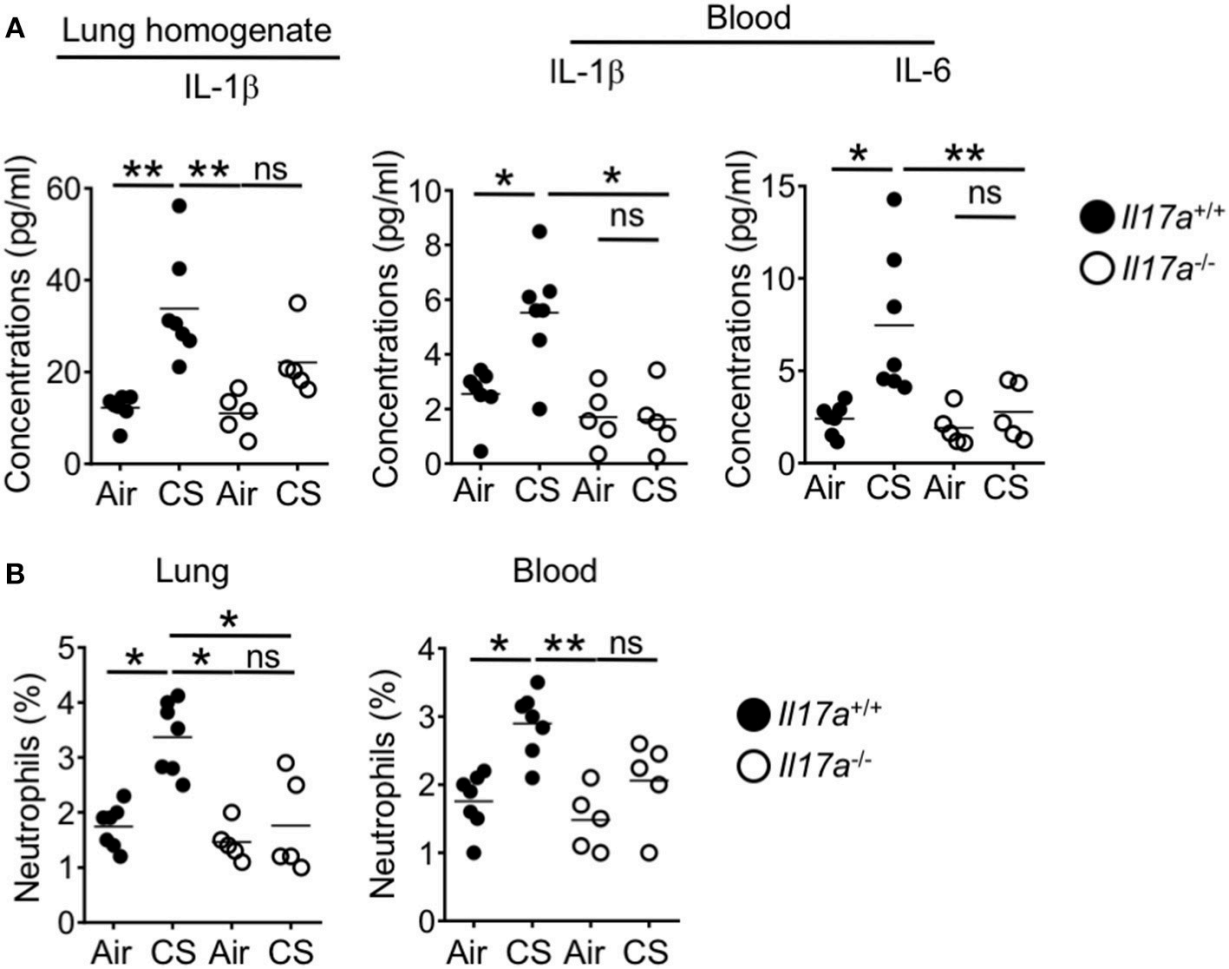
Increased lung IL-17A after CS exposure drives the systemic inflammation and neutrophilia in the lung and blood. IL-17A wildtype or knockout mice were exposed to air or smoke for 2 months. **(A)** Levels of pro-Th17 cytokine, IL-1β in the lung and IL-1β and IL-6 in the blood as determined by Luminex assay. **(B)** Frequencies of neutrophils (CD11b^+^Ly6G^+^ cells) in the lung and blood. Representative and pooled data obtained (mean ± SEM) from two independent experiments with 5–6 animals are shown. Kruskal-Wallis test with Dunn's multiple comparison was used to determine significance. ns, *P* > 0.05; ^*^P ≤ 0.05; ^**^P < 0.01. All mice are on the B6 background and were cohoused for 2 weeks before smoke exposure.

As we identified increased Th17 cells, ILC3 and neutrophil responses in the intestine after CS exposure during DSS-colitis, we next wanted to determine whether these immune signals drive the observed enhanced intestinal pathology. IL-17A deficient mice show reduced pathology in models of smoking induced emphysema (12). After short term CS exposure wildtype mice showed enhanced lung IL-1β as well as elevated IL-1β and IL-6 in the blood. This enhanced response was absent in IL-17 deficient mice (Figure 6A). As IL-17 induces neutrophil recruitment, we no longer observed enhanced recruitment of lung or blood neutrophils in IL-17A deficient mice after CS exposure (Figure 6B). This indicates that IL-17A drove the systemic inflammation including neutrophil recruitment into lung tissue and systemic neutrophilia in CS exposed mice.

**Figure 7:**
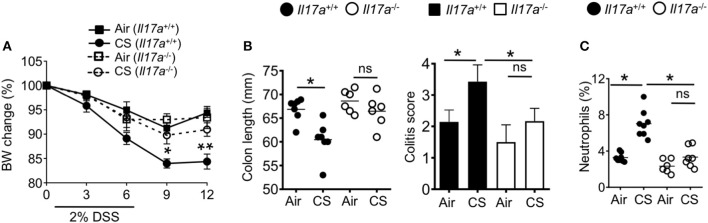
Cigarette smoke-induced neutrophil recruitment and intestinal inflammation depends on IL-17A. **(A)** Body weight change (%) of indicated mice exposed to air or CS for 2 months followed by DSS treatment. **(B)** Colon length and colitis scores after 2% DSS treatment. **(C)** Frequencies of neutrophils (CD11b^+^Ly6G^+^ cells) in the colon after DSS treatment were assessed by flow cytometry. Representative and pooled data obtained (mean ± SEM) from two independent experiments with 6–8 animals are shown. Kruskal-Wallis test with Dunn's multiple comparison were used to test for significance. ns, *P* > 0.05; ^*^P ≤ 0.05; ^**^P < 0.01.

To understand the role of IL-17A in CS-induced intestinal inflammation, we exposed *IL-17a*^−/−^ mice to CS followed by treatment with DSS. While wildtype mice showed increased weight loss and intestinal pathology after CS exposure (Figures 1, 7A,B) this increased pathology was lost in CS exposed IL-17A deficient mice (Figures 7A,B). Further, after DSS challenge, we did not find enhanced neutrophil numbers in the intestines of IL-17A deficient CS mice (Figure 7C). Collectively, increased lung IL-17A after CS exposure is responsible for increased neutrophil recruitment into the mucosal tissues.

### Neutrophils Mediate CS-Driven Intestinal Inflammation

**Figure 8:**
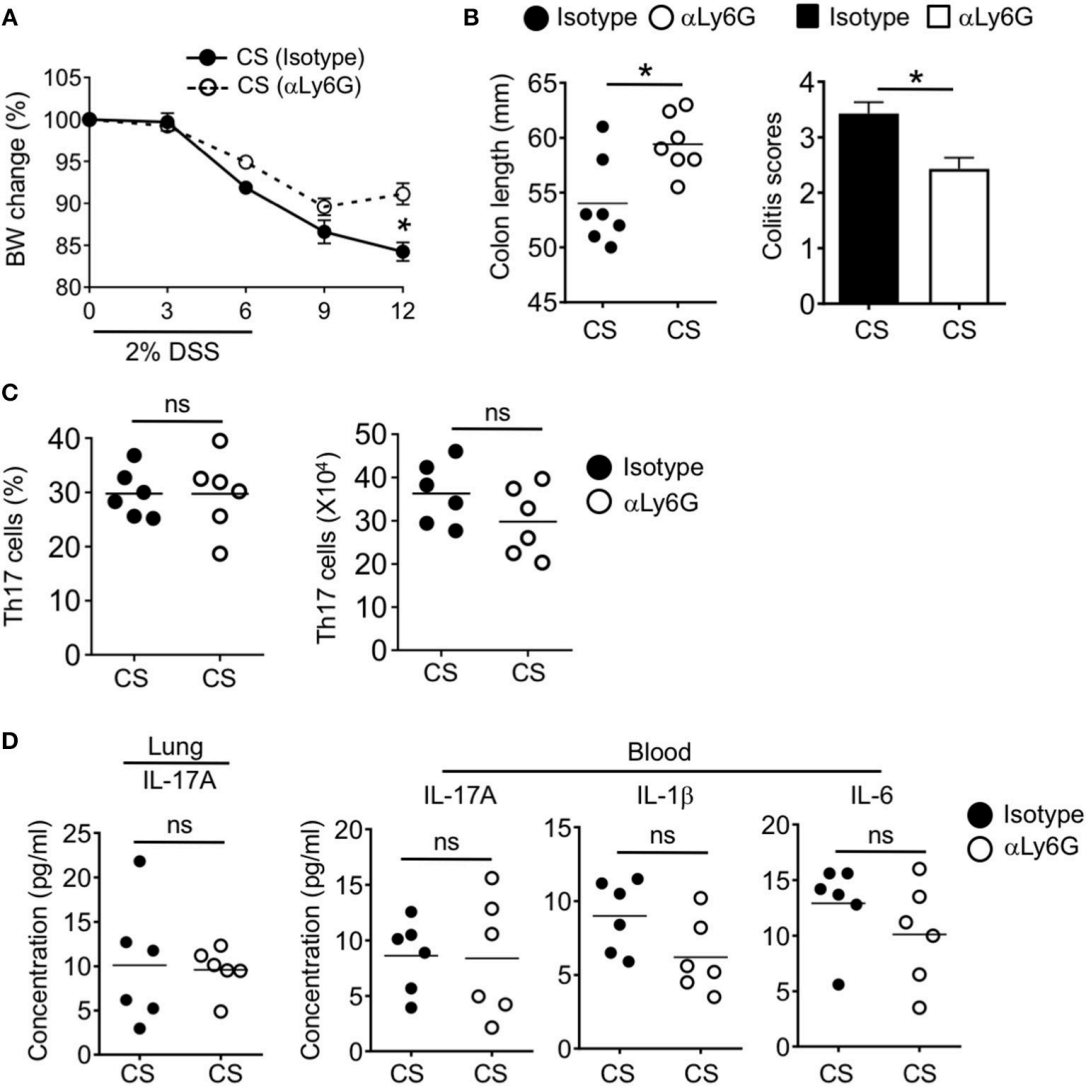
Neutrophils mediate cigarette smoke-enhanced intestinal inflammation. Two months CS exposed mice were treated with 2% DSS followed by treatment with isotype or neutrophil depleting αLy6G antibody at −2, 0, and 2 days post-DSS challenge. **(A)** Body weight change (%) and **(B)** colon length and colitis scores of CS mice with intact or depleted of neutrophils after DSS-induced acute inflammation. **(C)** Frequencies and numbers of Th17 cells in the intestine after DSS treatment were assessed by flow cytometry. **(D)** Quantification of Th17 cell response related cytokines in the lung homogenate and blood by Luminex assay. Data is representative of two independent experiments (mean ± SEM). Representative and pooled data obtained (mean ± SEM) from two independent experiments with 6–7 animals are shown. Mann-Whitney was used to test for significance. ns, *P* > 0.05; ^*^P ≤ 0.05.

We next wanted to determine if neutrophils were required for the observed increased intestinal pathology after CS exposure. We depleted neutrophils by injecting air and CS exposed mice with anti-Ly6G antibodies or isotype before and during DSS challenge (−2, 0, 2 days post-DSS). Mice treated with anti-Ly6G had reduced neutrophil frequencies throughout the body including in the colon ((Supplementary Figure 5). While we found a slight improvement in colitis score after neutrophil depletion of air treated mice, we found no alteration in weight loss, colon length or Th17 cell responses (Supplementary Figure 6). In contrast, neutrophil depletion protected CS exposed mice. Anti-Ly6G treated animals exhibited reduced weight loss, normalized colon length and improved colitis scores as compared to isotype treated CS exposed mice (Figures 8A,B). However, we still observed comparable increased frequencies and numbers of Th17 cells in the colon of CS treated mice with intact or depleted of neutrophils (Figure 8C). In CS/DSS mice, IL-17A remained elevated in the lung and blood after neutrophil depletion along with increased IL-1β and IL-6 in the blood (Figure 8D). Overall, CS exposure leads to local Th17 cell responses in the lung which drives increased circulating neutrophils and inflammatory cytokines. After intestinal injury, IL-17A drives increased intestinal pathology through increased neutrophil recruitment into intestinal tissue (Supplementary Figure 7).

## Discussion

In this study, we utilized an animal model of short-term smoke exposure to understand how lung inflammatory signals could impact pathological responses within intestinal tissue and identify early local and systemic changes after smoke exposure. After 2 months of CS exposure, we do not find signs of histological disease within the lung. However, we find elevated Th17 cell responses along with elevated pro-Th17 cell cytokines, such as IL-1β in the blood. However, we do not observe altered inflammatory changes in the intestine after CS exposure.

IL-17A is produced by Th17 cells and ILC3 and plays an important role in both induction of tissue inflammation, anti-microbial immunity, and colorectal cancer (20). These effects are partially mediated though recruitment of neutrophils into inflamed tissue (17). As predicted, CS exposure alone increases neutrophil numbers in the lung in parallel to increased IL-17A and Th17 cells. We also find elevated neutrophils in the blood of CS exposed mice however this does not extend to intestinal tissue as CS alone is insufficient to increase intestinal or MLN neutrophil numbers. Together, these data indicate that short term CS exposure may prime the systemic immune system but does not directly lead to increased tissue damage or inflammation within the intestine.

To ask if CS induced alterations in immune responses in the lung and circulation were sufficient to alter immune responses after intestinal damage, we utilized a combined mouse model of CS exposure and DSS-induced colitis. We found that CS exposure enhanced intestinal pathology. Alongside increased weight loss and colitis score, we also found increased intestinal numbers of Th17 cells and ILC3 in CS exposed mice undergoing DSS-induced colitis. Supporting a role for IL-17 induced neutrophil recruitment, we also found increased neutrophil numbers in the intestines of CS exposed mice after DSS treatment.

We next demonstrated that IL-17A is a critical mediator of enhanced intestinal inflammatory damage after CS exposure. In CS exposed mice, IL-17A deficiency reduced blood pro-Th17 cytokines IL-1β and IL-6 as well as circulating neutrophils to the levels found in air exposed mice. After DSS challenge, CS exposure did not increase intestinal inflammation in mice deficient for IL-17A with intestinal pathology and neutrophil numbers comparable to those found in Air exposed mice. This indicates that lung IL-17A induced by CS exposure is important for sensitizing systemic inflammation.

In line with our findings, other studies have reported increased lung and blood neutrophils in smokers (21, 22). Further, mouse models of CS exposure also report increased lung neutrophils (13). However, the role of CS-induced increased circulating neutrophils in inducing distal inflammation has not been determined. In this study, through depletion of neutrophils during intestinal inflammation, we demonstrated that neutrophil responses driven by CS play a significant role in enhanced intestinal inflammation after DSS challenge. Neutrophil depletion partially rescued increased intestinal inflammation in CS exposed mice. While neutrophils are the major downstream effector of IL-17, there are likely additional mediators downstream of the increased IL-17A as IL-17A deficiency more completely ameliorated CS-enhanced pathology. Together, these data suggest that the neutrophil response is downstream of lung IL-17A and Th17 cell responses driven by CS.

It is speculated that the mucosal immune system is itself a “system wide organ” in which the mucosal immune cells distributed throughout the body can communicate between or among different mucosal tissues or organs (7). However, the majority of studies of the mucosal immune system focus on understanding responses within individual organs. Recent studies have supported reciprocal regulation of immune responses between lung and intestinal tissue (23). For example, the gut microbial metabolites short chain fatty acids regulate lung immunity in a model of allergy (24, 25). Viral infection in the lung can induce intestinal injury through Th17 cell responses (26). However, many questions remain about how such cross talk is achieved or what immune signaling pathway transfers signals between the various mucosal sites. In this study, altered lung immunity after CS exposure promoted Th17 cell and downstream neutrophil responses in the intestine after DSS challenge. We further demonstrated that the Th17 cell and neutrophil responses are major immune pathways for communicating between the lung and intestine.

Intestinal factors, such as the microbiota also likely influence this axis. Within the mouse intestine, there are examples of microbes that can drive local immunity that is either pro- or anti-inflammatory. We and others have identified intestinal microbes that can activate local anti-inflammatory responses (27, 28). Segmented filamentous bacteria (SFB) is a mouse commensal that induces local Th17 cell responses and can also sensitize to both local and distal inflammation (29, 30). Our mice are SFB free and so have limited steady state intestinal Th17 cell responses. It will be interesting to understand if intestinal microbiota alterations further sensitize to or can protect from lung Th17 responses.

Together, we find intrinsic or extrinsic factors that alter the circulating neutrophil pool and/or recruitment of neutrophils to tissue sites after immune challenge are a cellular mechanism of immune regulation between distal tissues. Our studies demonstrate a critical role for this Th17-neutrophil axis in driving intestinal inflammation after CS exposure. Our work also provides evidence that communication between individual arms of the mucosal immune system can contribute to increased pathology in disease.

## Author Contributions

GD and MK designed and analyzed experiments and wrote the manuscript with input from all co-authors. MK and BG performed and analyzed the experiments. MM, HS, KN, AH performed experiments. DC, W-JW, and FK designed and analyzed the experiments.

### Conflict of Interest Statement

The authors declare that the research was conducted in the absence of any commercial or financial relationships that could be construed as a potential conflict of interest.
