# Supplementary material for: Cigarette Smoke Induces Intestinal Inflammation via a Th17 Cell-Neutrophil Axis
Source: Front Immunol. 2019 Jan 29;10:75. doi: 10.3389/fimmu.2019.00075 (PMC6361762; doi:10.3389/fimmu.2019.00075)
Supplement: Supplementary file 1 [file Data_Sheet_1.pdf]

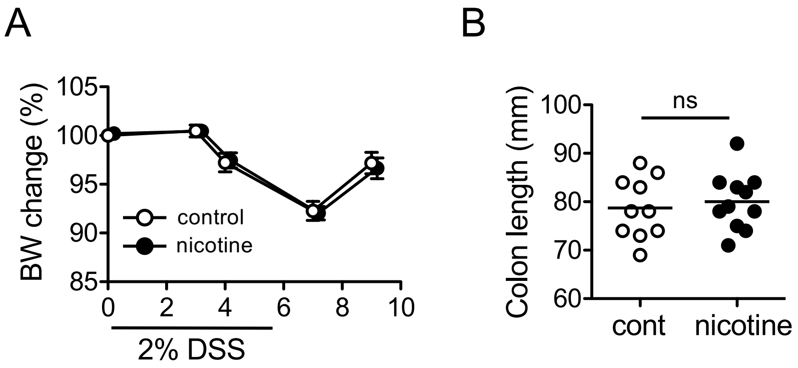

S. Figure 1.Nicotine does not alter mouse sensitivity to DSS. Mice were exposed to vehicle or nicotine by intranasal administration before treatment with 2% DSS for 6 days. Body weight changes (A), colon length (mm) (B). Representative and pooled data obtained (mean ± SEM, n=8-11) from 2 independent experiments are shown. T-test or repeated measure ANOVA was used to determine significance.

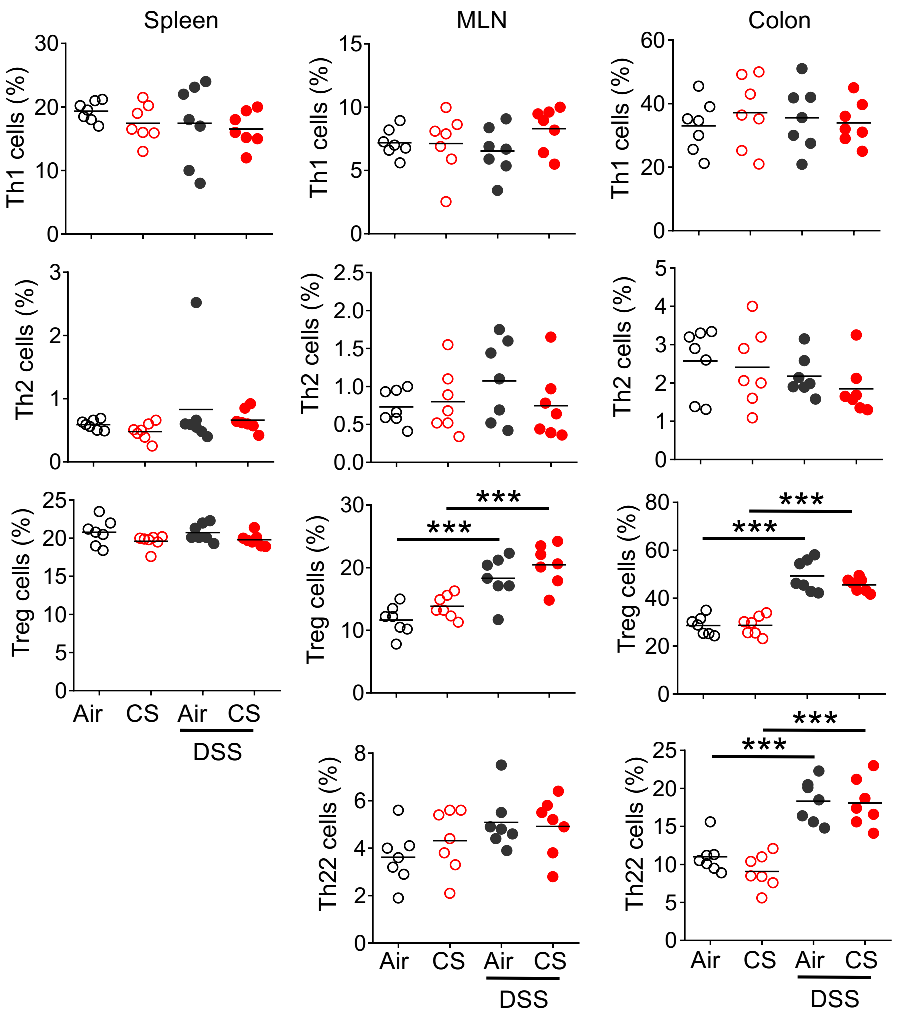

S. Figure 2. Cigarette smoke does not impact T cell subsets outside the lung. Mice were treated with air or CS exposure followed by water or DSS for 7 days. Frequencies of Th1 (T-bet^{+}CD4^{+}), Th2 (Gata3^{+}CD4^{+}), Treg (FoxP3^{+}CD4^{+}), Th22 (IL-22^{+}CD4^{+}) cells in spleen, MLN, or colonic lamina propria were examined by flow cytometry. Representative data obtained from 2 independent experiments are shown. ANOVA with Bonferroni's correction was used to test for significance. ns, P>0.05, ***, P < 0.001.

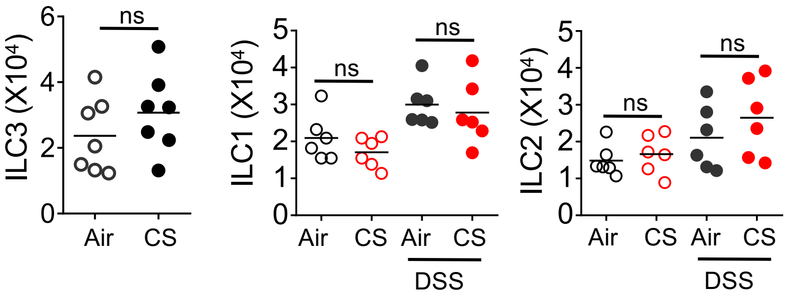

S. Figure 3. Cigarette smoke does not directly impact intestinal ILC subsets. Mice were treated with air or CS exposure followed by water or DSS. Absolute numbers of ILC3 (Lineage$^-$CD90$^+$T-bet$^-$RORγt$^+$ cells) ILC1 (Lineage$^-$CD90$^+$T-bet$^+$RORγt$^+$ cells), and ILC2 (Lineage$^-$CD90$^+$gata3$^+$ cells) in colonic lamina propria were examined by flow cytometry. ILC3 numbers with DSS treatment are shown in Figure 5D. Representative data obtained from 2 independent experiments are shown. Student's T test or ANOVA with Bonferroni’s correction was used to test for significance.

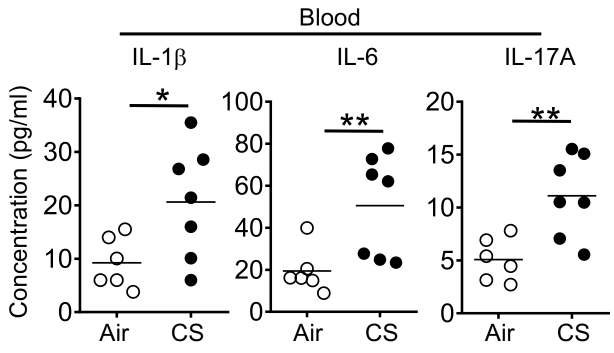

S. Figure 4. Increased blood Th17 related cytokines in blood of CS exposed DSS treated mice. Mice were exposed to air or CS for 2 months followed by 2% DSS for 6 days. Mice were sacrificed at 9 days post DSS treatment. Quantification of indicated cytokines in the blood from Air and CS mice after DSS challenge as determined by Luminex assay. Significance was determined by Mann-Whitney test. *, $P \leq 0.05$; **, $P < 0.01$

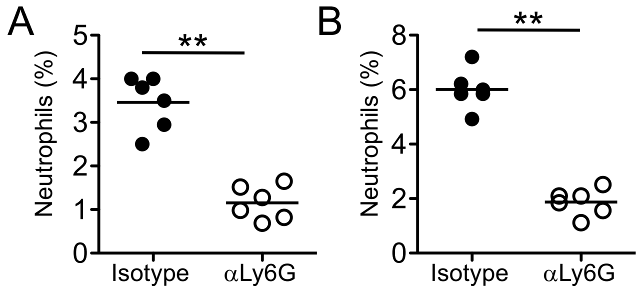

S. Figure 5. Neutrophil depletion with αLy6G antibody. 2 month Air (A) or CS (B) exposed mice were treated with 2% DSS followed by treatment with isotype or neutrophil depleting αLy6G antibody at -2, 0, and 2 days post DSS challenge. Neutrophil frequencies in the colon was determined by flow cytometry. Mann-Whitney was used to test for significance. **, $P < 0.01$.

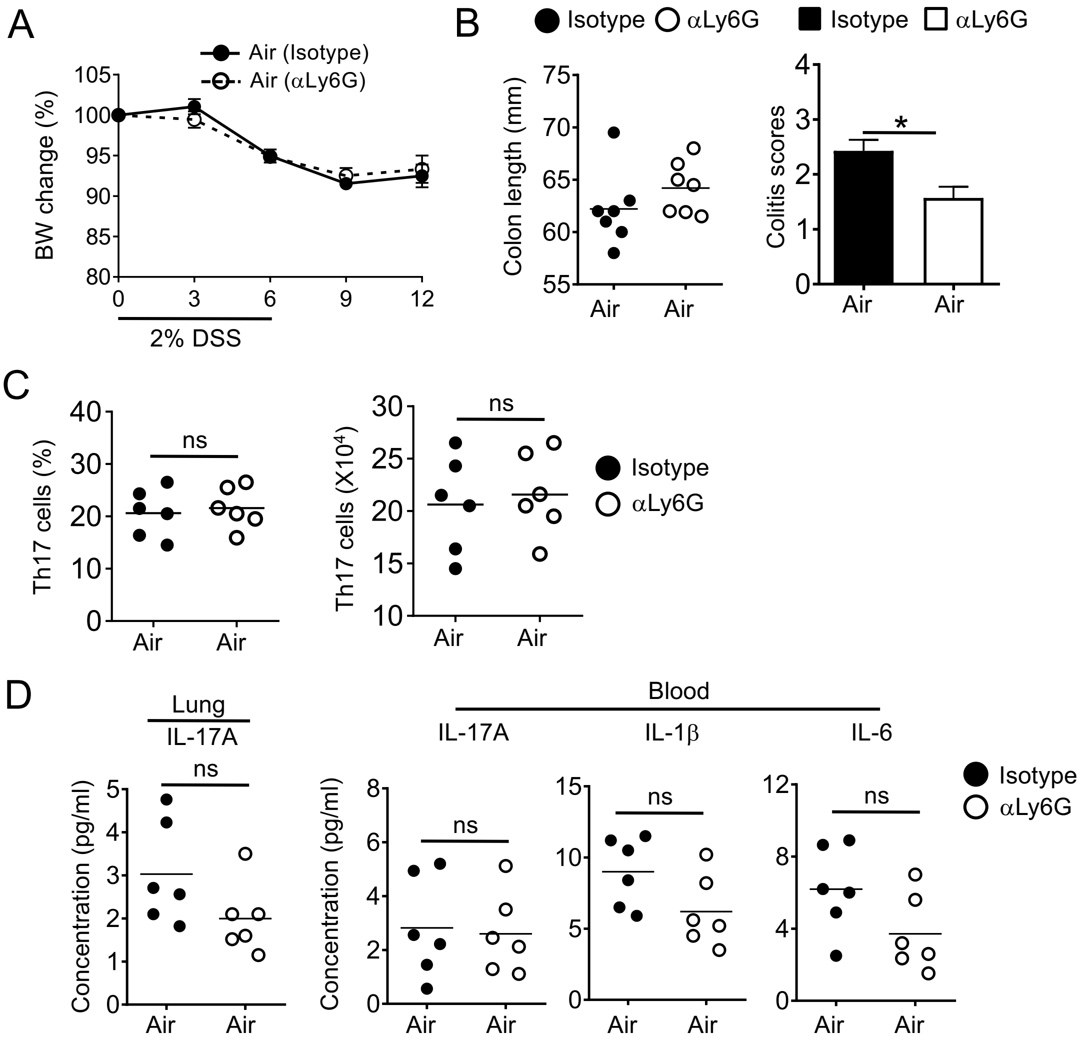

S. Figure 6. Neutrophils mediate cigarette smoke-enhanced intestinal inflammation. (A) Body weight change (%) of Air treated mice treated with αLy6G antibody or isotype control at -2, 0, and 2 days post DSS challenge. (B) Colon length and colitis scores after 2% DSS treatment in mice described above. (C) Frequencies and numbers of Th17 cells in the intestine after DSS treatment were assessed by flow cytometry. (D) Quantification of Th17 cell response related cytokines in the lung homogenate and blood by Luminex assay. Representative and pooled data obtained (mean ± SEM) from 2 independent experiments with 6-7 animals are shown. Repeated measured ANOVA with pairwise comparisons on day 12 with Bonferonni adjustments for multiple comparisons (A) or Mann-Whitney (B-D) were used to test for significance. ns, P>0.05; *, P ≤0.05; **, P < 0.01.

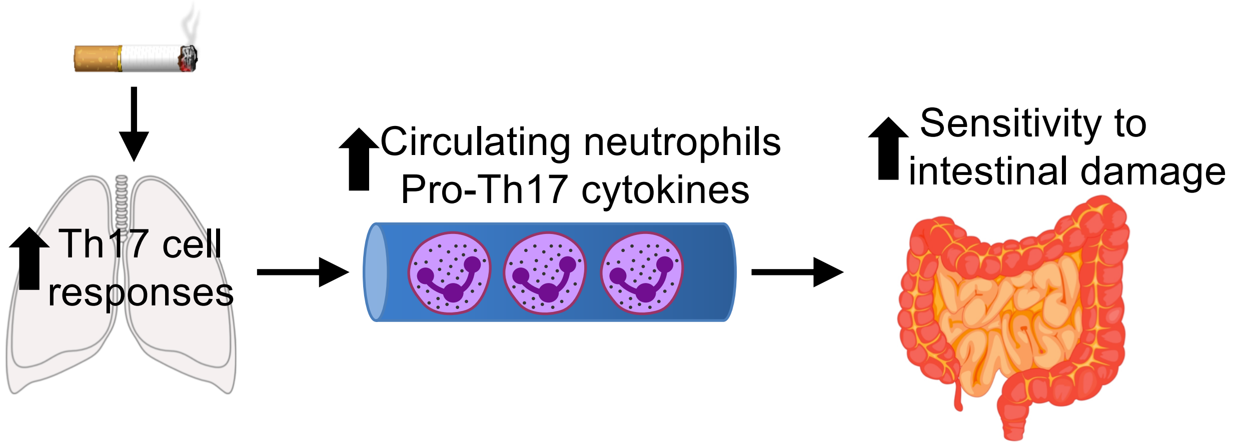

S. Figure 7. Cigarette smoke exposure leads to a local Th17 response in the lung. This leads to increased circulating neutrophils and pro-Th17 cytokines that can sensitize to intestinal damage
